# Burden of gluteal fibrosis and post-injection paralysis in the children of Kumi District in Uganda

**DOI:** 10.1186/s12891-018-2254-9

**Published:** 2018-09-24

**Authors:** Kristin Alves, Norgrove Penny, John Ekure, Robert Olupot, Olive Kobusingye, Jeffrey N. Katz, Coleen S. Sabatini

**Affiliations:** 1 Harvard Combined Orthopaedic Surgery Residency Program, Boston, MA USA; 2 Department of Orthopaedic Surgery, University of British Colombia, Victoria, Canada; 3 Kumi Orthopaedic Center, Kumi, Uganda; 4 Kumi Hospital, Kumi, Uganda; 5 Makerere University School of Public Health, Kampala, Uganda; 6 Department of Orthopaedic Surgery, Brigham and Women’s Hospital, Boston, MA USA; 7 University of California San Francisco Department of Orthopaedic Surgery, UCSF Benioff Children’s Hospital Oakland, 747 52nd Street, OPC 1st Floor, Oakland, CA 94609 USA

**Keywords:** Gluteal fibrosis, Post-injection paralysis

## Abstract

**Background:**

The purpose of this study was to estimate the prevalence of postinjection paralysis (PIP) and gluteal fibrosis (GF) among children treated in a rural Ugandan Hospital.

**Methods:**

We conducted a retrospective cohort study by reviewing the musculoskeletal clinic and community outreach logs for children (age < 18 yrs) diagnosed with either PIP or GF from Kumi Hospital in Kumi, Uganda between 2013 and 2015. We estimated the prevalence as a ratio of the number of children seen with each disorder over the total population of children seen for any musculoskeletal complaint in musculoskeletal clinic and total population of children seen for any medical complaint in the outreach clinic.

**Results:**

Of 1513 children seen in the musculoskeletal clinic, 331 (21.9% (95% CI 19.8–24.1%)) had PIP and another 258 (17.1% (95% CI 15.2–19.0%)) had GF as their diagnosis. Of 3339 children seen during outreach for any medical complaint, 283 (8.5% (95% CI 7.6–9.5%)) had PIP and another 1114 (33.4% (95% CI 31.8–35.0%)) had GF. Of patients with GF, 53.9% were male with a median age of 10 years (50% between 7 and 12 years old). Of patients with PIP, 56.7% were male with a median age of 5 years (50% between 2 and 8 years old).

**Conclusion:**

PIP and GF comprise over 30% of clinical visits for musculoskeletal conditions and 40% of outreach visits for any medical complaint in this area of Uganda. The high estimated prevalence in these populations suggest a critical need for research, treatment, and prevention.

## Background

Approximately 12 billion intramuscular injections are administered annually worldwide for a wide range of conditions, with persons in limited-resource countries receiving an average two injections per year [1]. Over 70 % of these injections are estimated to be unnecessary [2]. Disabilities arising from injections include acute flaccid paralysis after injection due to sciatic nerve injury, also known as post-injection paralysis, and gluteal fibrosis [3–12]. Both of these entities have been reported among children in Uganda [4, 11].

When gluteal intramuscular injections are misdirected into the sciatic nerve or neurotoxic medications are delivered near the nerve, children may develop post-injection paralysis (PIP) acutely with loss of motor and sensory function of the sciatic nerve distal to the injection. The strong temporal association between the injections and subsequent neurologic findings argues for a causal relationship between injection and PIP. Children with post-injection paralysis present with varying degrees of the initial foot drop and the later acquired equino-varus foot deformities depending on timing of their presentation to the health care system [6–9, 11, 13, 14].

The etiology of gluteal fibrosis (GF) has not yet been determined; hypotheses range from a congenital collagen disorder to iatrogenic injection injury. The most commonly reported hypothesis involves frequent gluteal intramuscular injections with the percentage of patients with gluteal fibrosis in the literature reporting a history of gluteal injections ranging from 51 to 100%. [3–5, 9, 10, 15, 16]. GF is characterized by hypertrophy of fibrotic tissue in the gluteal muscles that limits muscle excursion and therefore, hip range of motion. It was recognized and described in the 1970s [16, 17]. Patients with gluteal fibrosis present with difficulty with activities like squatting and sitting normally in a chair because they have limited excursion of the gluteal muscles causing external rotation and abduction when the hips are flexed actively or passively. On examination, patients are often found to have fixed abduction and external rotation with attempts at active or passive hip flexion. They have an awkward gait due to the deranged biomechanics raising concern for their risk of premature osteoarthritis [23]. The condition is usually bilateral and frequently diagnosed in school age children [3, 18, 19]. GF can limit the patient’s ability to attend school and perform activities of daily living and community activities.

GF has been reported increasingly in Asia, Europe, Africa, and the USA [3–5, 9, 10, 12, 15–24]. However, few studies have assessed the prevalence of either PIP or GF in a particular, localized area. Published estimates for GF generally range between 1 and 2.5% in affected populations, with prevalence seen as high as 13.9% of the pediatric population in some districts of Taiwan [3, 12, 15, 18, 19, 22]. Both PIP and GF are seen commonly throughout Uganda, necessitating considerable care, yet the prevalence of these injuries in Uganda or elsewhere in East Africa has not been quantified, impeding efforts to estimate resource needs for these conditions [4, 8, 11]. Thus, our study aims to determine prevalence for GF and PIP in the pediatric population presenting for care in Kumi, a northeastern district of Uganda anecdotally known to have cases of GF and PIP.

## Methods

### Design

This was a retrospective cohort study of all pediatric children seen in Kumi Hospital’s musculoskeletal clinic and in Kumi District’s general medical outreach clinic logbooks between January 2013 and December 2015.

### Setting

Kumi Hospital is a rural hospital located in Kumi District and serves the district’s population through inpatient, outpatient and outreach services. Kumi Hospital has a musculoskeletal clinic located at the hospital for outpatients with musculoskeletal problems. The hospital also runs outreach programs held in outlying villages where patients are seen with a wide range of problems. The study was approved by Makerere Institutional Review Board in Kampala, Uganda and the Uganda National Council for Science and Technology as well as the senior author’s (CSS) institution.

### Sample

The study sample included children (age ≤ 18 yrs) diagnosed with either PIP or GF from Kumi Hospital musculoskeletal clinic or Kumi District outreach medical visits between January 2013 and December 2015. Only children labeled as a new visit were recorded. PIP was diagnosed as initial acute flaccid paralysis after gluteal injection with later development of equinovarus foot deformity. GF was diagnosed as gluteal contractures causing limitation of hip range of motion, specifically obligate external rotation and abduction with hip flexion, with no other known cause.

### Data sources and data elements

All patient information was obtained from written logbooks. The Kumi Hospital clinic logbooks included only patients seen in the clinic for musculoskeletal complaints (including pain, disability, deformity, or injury involving any extremity or the spine). The Kumi District Outreach visit logbooks included patients seen for any medical reason throughout Kumi District. For each set of logbooks, only patients ages 0–18 were collected for analysis. Data collected included patient’s age, sex, village, diagnosis, and recommendation for treatment.

### Reliability of data collection

Intraobserver reliability was assessed to ensure accuracy in data collection and data entry. KA selected 250 patients randomly and compared all variables entered into the database against the original log book entries; 100% of the variables matched initial entry. To determine interobserver reliability, a separate reviewer repeated the data abstraction process (from written logbook to Excel) on 100 randomly selected subjects. The two observers were in agreement on 99.7% of all data abstracted.

Furthermore, to ensure there was no overlap in patients seen with GF and PIP from the Kumi hospital clinic and Outreach logbooks, cross-checks between logbooks were performed. This process involved selecting groupings of GF and PIP patients seen in Outreach and cross checking Clinic logbooks to see if the same patient names showed up within a 3 month period after they were seen in Outreach. This process was performed for 200 patients with GF or PIP, with no evidence of children having been counted as a new patient for both Clinic and Outreach visits.

### Data management

Once the logbook data was abstracted, patients’ diagnoses and treatment recommendations were coded. Codes for diagnosis included: gluteal fibrosis, post-injection paralysis, both disorders, and other medical conditions. Codes for treatment recommendation included: surgery (only applicable for clinic visits), referral to surgeon (only applicable for outreach visits), physical therapy, assistive devices (i.e. ankle foot orthoses (AFOs)), and other recommendations (e.g. further testing including xrays, medications, etc.). The accuracy of these diagnosis codes was verified in a process in which the Principal Investigator examined the agreement between the diagnosis code and the actual diagnoses listed for every 4th patient entered in the database. The codes and original listed diagnosis agreed in 100% of the patients reviewed.

### Statistical analysis

Statistical analysis was conducted using SAS 9.4 (SAS, Cary, NC, USA). For the clinic population, we estimated the prevalence of PIP, as a ratio of the number of children with a PIP diagnosis divided by the total population of children seen for any musculoskeletal complaint. For the community outreach population, we estimated the prevalence as the number of children with a PIP diagnosis divided by the total population of children seen for any medical complaint. The clinic and outreach prevalences of GF were estimated analogously. We used descriptive statistics (mean, median) to examine patient factors including sex, age, location and treatment recommendation. 95% confidence intervals were calculated assuming a binomial distribution. The median age of patients with PIP and those with GF were compared utilizing the Wilcoxon signed rank test. A *p* value of less than 0.05 was considered statistically significant. Treatment recommendations were determined and reported for each clinical setting.

## Results

Of the 4852 total children, 1372 were diagnosed with gluteal fibrosis (28.3% (95% CI 27.0–29.6%)) and 614 were diagnosed with post-injection paralysis (12.7% (95% CI 11.8–13.6%)) (Table 1). Only 15 of these children were found to have both disabilities (0.3%). Of 1513 children seen in the musculoskeletal clinic, 331 (21.9% (95% CI 19.8–24.1%)) had PIP and another 258 (17.1% (95% CI 15.2–19.0%)) had GF as their diagnosis. Of 3339 children seen on outreach in the community for any medical complaint, 283 (8.5% (95% CI 7.6–9.5%)) had PIP and another 1114 (33.4% (95% CI 31.8–35.0%)) had GF as their diagnosis. The diagnosis of another fibrotic disease, quadriceps fibrosis (QF), was made in 46 children (1%).

**Table 1:** Demographic Distribution

	No (%)	Sex (% male)	Age (median, years) (25th – 75th percentile, years)
GF	1372 (28.3)	53.9	10 (7–12)
Outreach	1114	53.4	10 (7–12)
Clinic	258	55.8	10 (8–12)
PIP	614 (12.7)	56.7	5 (2–8)
Outreach	283	52.7	6 (3–9)
Clinic	331	60.1	4 (2–8)
Other	2881 (59.4)	54.6	6 (3–11)

Demographic distribution of Gluteal Fibrosis (GF) and Post-Injection Paralysis (PIP) in Kumi District, Uganda. Of note, 15 patients had GF and PIP diagnosis, 8 in outreach and 7 in clinic

**Fig. 1:**
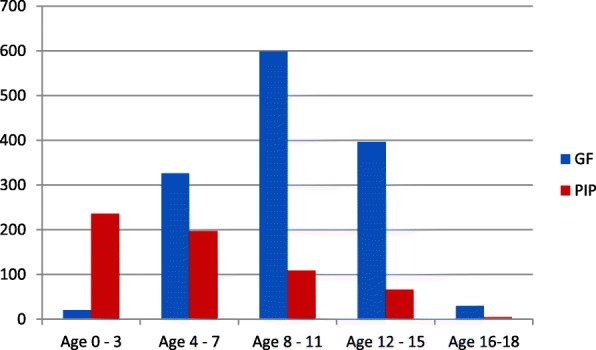
Case Distribution by Age group. PIP was found more often in children under age 8 while GF more often found in children older than age 8. (*p-value < 0.0001 for association between age and diagnosis, Wilcoxon test)

For both GF and PIP, a larger proportion was male; this gender difference was greater in the clinic population (Table 1). The median age for the GF patients was 10 years (25th – 75th percentile 7–12 years) while the median age for the PIP patients was 5 (25th – 75th percentile 2–8 years) (Fig. 1). The prevalence of GF increased with age, peaking at age group 8–11. In contrast, PIP prevalence was highest in the younger children with a peak in 0–3 and declining thereafter (Fig. 1).

The majority of the children seen with GF were recommended to have release if seen in clinic (92.6%) and referral to a surgeon if seen on outreach visits (87.1%). In both the clinic and outreach settings, recommendations for PIP generally consisted of non-operative management including AFOs and physiotherapy (Table 2).

**Table 2:** Distribution of Treatment Recommendations

	No (%)	Surgery	Referral to Surgeon	Assistive Device	Physical Therapy	Other
Outreach Patients	3339					
GF	1114 (33.4)	n/a	970 (87%)	0	133 (12%)	11 (1%)
PIP	283 (8.5)	n/a	92 (33%)	109 (39%)	55 (19%)	27 (10%)
Clinic Patients	1513					
GF	258 (17.1)	239 (93%)	n/a	0	14 (5%)	5 (2%)
PIP	331 (21.9)	44 (13%)	n/a	151 (46%)	118 (36%)	18 (5%)

Distribution of treatment recommendations for Gluteal Fibrosis (GF) and Post-Injection Paralysis (PIP) in Kumi District, Uganda

## Discussion

This retrospective review of all pediatric patients seen over a 3-year span in a northeastern Ugandan musculoskeletal clinic and comprehensive community-based rehabilitation outreach program highlights the community burden of both PIP and GF. These disabilities were found to comprise over 30% of hospital clinic visits for musculoskeletal conditions and 40% of outreach visits for any medical complaint in this northeastern region of Uganda.

The slightly greater proportion of males with both diagnoses may reflect an increased propensity to seek care for male children, a trend noted in other cultures [3]. In this rural region of Uganda, traveling to clinic generally takes more time and resources than an outreach visit near the child’s village. We found a correspondingly greater percent of children to be male in the clinic setting in comparison to the outreach setting.

The current hypothesis for the etiology of PIP and GF, and for the greater prevalence in this study than the 1–13.9% reported in other countries, revolves around the treatment of malaria [3, 15, 18, 19, 22]. Commonly in East Africa, infants and young children who develop high fevers are thought to be suffering from malaria. Patients’ families seek care at various locations including local traditional healers, local clinics, hospitals, outreach clinics, pharmacies, etc. At one or another of these providers, children may receive a gluteal injection of medication and can develop acute PIP if the drug is neurotoxic or if the sciatic nerve is damaged directly. One prior assessment at Mulago Hospital in the capital of Uganda highlights this association, with 70% of the gluteal injections that resulted in PIP being administered for malaria or febrile illness. The medication quinine was involved in approximately 60% of those cases [8, 11]. Quinine is neurotoxic and can cause neural injury by being injected in the vicinity of the nerve [11]. It does not have to be injected into the nerve directly as it causes tissue necrosis. This neurotoxic intramuscular medication is presumably being given to children with suspected or diagnosed malaria although it is not recommended as a first line anti-malarial by the WHO [25].

While these practices help explain the potential cause of PIP in Uganda, little is known about the etiology and risk factors for gluteal fibrosis. The difficulty in determining the etiology of GF stems from the lack of a close temporal association with injections. Hang et al. and Napiontek et al. observed that GF developed two to 5 years after the damaging injections [9, 16]. This finding is consistent with our finding that children with GF averaged 10 years of age compared to 5 for children with PIP. Confirming the involvement of injections in the pathogenesis of GF, Ko et al. and Chung et al. note associations between intramuscular injections and gluteal fibrotic contractures in their case control studies with increasing odds of GF with increasing frequency of injections [3, 5]. The mechanisms of injury following injection have been hypothesized to include sterile abscesses, compartment syndrome caused by large volume injection, and muscle necrosis caused by toxic medications or toxic diluting solvents [12, 15, 21, 22]. Regardless of the cause, the common theory is that the fibrotic bands develop over time leading to clinical contracture at a later age.

Some authors further hypothesize a predisposing collagenous risk factor for gluteal fibrosis, with the intramuscular injection acting as a trigger mechanism [5, 16]. This intriguing theory finds some support in Uganda. While injection induced injuries (PIP) have been seen throughout the country, GF has been reported primarily in northeast Uganda, suggesting that an additional exposure may be acting as an effect modifier of the injections in this region. Further studies are underway to elucidate country wide prevalence and distribution of PIP versus GF, potential risks for development of these injection injuries to validate this claim and determine the focus of resources for prevention and treatment outcomes.

The current study demonstrates a large population of children in northeast Uganda suffering from PIP and GF with significant resource needs both for operative interventions and nonoperative (ie. AFOs and physical therapy) treatment. In addition to the significant proportion of children with PIP and GF, 46 children with quadriceps fibrosis were also seen, demonstrating even further potential injection-induced injuries and need for resources focused on injection induced disabilities and prevention education.

Our study must be interpreted in the context of methodologic limitations including retrospective data collection with a lack of data regarding potential sources of etiology of pathology, confirmation of and inclusion of only one region in Uganda. Another limitation of the study is the possibility of misdiagnosis as there are not confirmatory tests for these conditions outside the history and physical examination that are available in this environment. However, given the frequency of these conditions in the population, clinicians are very skilled at recognizing these findings on examination and when combined with relevant history questions, the diagnosis of PIP and GF can be confidently made. However, this study clearly demonstrates that a large proportion of pediatric patients seen for medical care are suffering from these disabilities in this region and supports the need for future research to determine the scope of the problem in Uganda. Further research should be focused on country-wide prevalence and incidence of GF and PIP, both quantitative and qualitative community-based studies to determine etiology and risk factors, as well as treatment studies to better understand how to care for children with these conditions.

While this study has focused on Uganda, prior studies have demonstrated sciatic nerve injury following injection in high and low resource countries alike [6, 7, 14]. To prevent further iatrogenic injuries it is imperative that countries begin to employ strict standards for injection education and delivery. In addition, it is important for orthopaedic surgeons to be aware of these diagnoses as noted by Scully et al. [10]. The unfamiliarity can lead to potential inaccurate diagnosis and treatment with children undergoing inappropriate operations with need for subsequent repeat surgery to correct the deformity. Resources for efforts to increase awareness and prevent further disability should be prioritized in addition to resources for the treatment of the current pediatric population suffering from these disabilities.

## Conclusion

This study documents a concerning prevalence of PIP and GF in one area of Uganda and suggests an urgent need for more rigorous studies of country-wide prevalence, incidence, prevention and treatment outcomes. While population-based estimates of disease burden are sorely needed, the high estimated prevalence of these conditions in these clinical populations in Kumi, Uganda, suggest that substantial resources are needed to address these conditions currently. Simultaneously, appropriate policy and health systems changes are required to ensure appropriate health education and administration of injections to prevent further burden of injection-induced diseases in the pediatric population.

## Abbreviations

GF: Gluteal Fibrosis
PIP: Post-injection paralysis
